# A longitudinal study on quality of life along the spectrum of Alzheimer’s disease

**DOI:** 10.1186/s13195-022-01075-8

**Published:** 2022-09-15

**Authors:** Arenda Mank, Judith J. M. Rijnhart, Ingrid S. van Maurik, Linus Jönsson, Ron Handels, Els D. Bakker, Charlotte E. Teunissen, Bart N. M. van Berckel, Argonde C. van Harten, Johannes Berkhof, Wiesje M. van der Flier

**Affiliations:** 1 Alzheimer Center Amsterdam, Neurology, Vrije Universiteit Amsterdam, Amsterdam UMC location VUmc, De Boelelaan 1118, 1081 HZ Amsterdam, The Netherlands; 2 Amsterdam Neuroscience, Neurodegeneration, Amsterdam, The Netherlands; 3 Amsterdam UMC, Vrije Universiteit Amsterdam, Department of Epidemiology and Data Science, Amsterdam Public Health Institute, Amsterdam, The Netherlands; 4 Department for Neurobiology, Care Sciences and Society, Division of Neurogeriatrics, Karolinska Institutet, Solna, Sweden; 5 Department of Psychiatry and Neuropsychology, Alzheimer Centre Limburg, School for Mental Health and Neurosciences, Maastricht University, Maastricht, The Netherlands; 6 Neurochemistry Laboratory and Biobank, Department of Clinical Chemistry, Amsterdam Neuroscience, VU University Medical Center Amsterdam, Amsterdam UMC, Amsterdam, The Netherlands; 7 Department of Radiology & Nuclear Medicine Amsterdam Neuroscience Vrije Universiteit Amsterdam, Amsterdam UMC, Amsterdam, The Netherlands

## Abstract

**Background:**

Quality of life (QoL) is an important outcome from the perspective of patients and their caregivers, in both dementia and pre-dementia stages. Yet, little is known about the long-term changes in QoL over time. We aimed to compare the trajectories of QoL between amyloid-positive and amyloid-negative SCD or MCI patients and to evaluate QoL trajectories along the Alzheimer’s disease (AD) continuum of cognitively normal to dementia.

**Methods:**

We included longitudinal data of 447 subjective cognitive decline (SCD), 276 mild cognitive impairment (MCI), and 417 AD dementia patients from the Amsterdam Dementia Cohort. We compared QoL trajectories (EQ-5D and visual analog scale (VAS)) between (1) amyloid-positive and amyloid-negative SCD or MCI patients and (2) amyloid-positive SCD, MCI, and dementia patients with linear mixed-effect models. The models were adjusted for age, sex, Charlson Comorbidity Index (CCI), education, and EQ-5D scale (3 or 5 level).

**Results:**

In SCD, amyloid-positive participants had a higher VAS at baseline but showed a steeper decline over time in EQ-5D and VAS than amyloid-negative participants. Also, in MCI, amyloid-positive patients had higher QoL at baseline but subsequently showed a steeper decline in QoL over time compared to amyloid-negative patients. When we compared amyloid-positive patients along the Alzheimer continuum, we found no difference between SCD, MCI, or dementia in baseline QoL, but QoL decreased at a faster rate in the dementia stage compared with the of SCD and MCI stages.

**Conclusions:**

QoL decreased at a faster rate over time in amyloid-positive SCD or MCI patients than amyloid-negative patients. QoL decreases over time along the entire AD continuum of SCD, MCI and dementia, with the strongest decrease in dementia patients. Knowledge of QoL trajectories is essential for the future evaluation of treatments in AD.

**Supplementary Information:**

The online version contains supplementary material available at 10.1186/s13195-022-01075-8.

## Introduction

The estimated number of patients with Alzheimer’s disease (AD) has increased tremendously over the past decades, and is projected to increase almost 3-fold in the next three decades [1]. In 2021, the estimated worldwide number of patients with dementia was 55 million, of which 60–70% have AD [2]. There is an even larger number of patients with pre-dementia stages of AD, although precise estimates are lacking.

Disease-modifying treatments have the potential to ameliorate the disease trajectory of AD and decrease the health burden on patients, caregivers, and society [3, 4]. Ultimately, the goal of treatments in AD, whether pharmaceutical, by (lifestyle) prevention or in terms of adjusting care, is to improve health-related quality of life (QoL). QoL reflects the impact of disease and treatment on physical, mental, social, and emotional well-being [5]. There is a variety of instruments to measure QoL, including the European Quality of Life-5 Dimensions (EQ-5D) which is widely used in research [6]. In addition, the visual analogue scale (VAS) may be more sensitive to show differences in trajectories of QoL between different types of dementia and controls [7, 8].

In an earlier study, we showed that the trajectory of QoL of patients is an important outcome from the perspective of patients and their caregivers, in both dementia and pre-dementia stages [9]. A recent review of QoL in individuals with normal cognition, MCI, and AD dementia identified several gaps in literature [6]. First, knowledge on QoL in the (biomarker-confirmed) pre-dementia stages is essential, because disease-modifying treatments increasingly focus on the pre-dementia stages in AD. Second, longitudinal studies are lacking, yet necessary to determine the long-term changes of QoL over time.

A recent cross-sectional study among biomarker-confirmed AD patients in the SCD and MCI stages showed no difference in the EQ-5D utilities between amyloid-positive and amyloid-negative individuals with subjective cognitive decline (SCD) and a higher EQ-5D utility in amyloid-positive mild cognitive impairment (MCI) patients compared to amyloid-negative MCI [10]. However, the cross-sectional nature of this former study does not allow insight in the trajectory of QoL over time in individuals.

In the current study, we aimed to investigate (1) the trajectory of QoL in amyloid-positive pre-dementia patients with SCD and MCI compared to amyloid-negative patients and (2) to evaluate the QoL trajectories along the spectrum of AD, i.e., amyloid-positive patients with SCD, MCI and dementia.

## Methods

### Participants

In this longitudinal study, we included *n* = 1140 patients from the Amsterdam Dementia Cohort (ADC). These included *n* = 105 amyloid-positive SCD, *n* = 342 amyloid-negative SCD, *n* = 144 amyloid-positive MCI, *n* = 132 amyloid-negative MCI, and *n* = 417 amyloid-positive dementia patients. All patients presented with complaints at the memory clinic of the Alzheimer center Amsterdam had their baseline visit between 2009 and 2020. Inclusion criteria were (1) a baseline diagnosis of Alzheimer’s disease dementia (AD), mild cognitive impairment (MCI), or subjective cognitive decline (SCD), (2) availability of amyloid PET and/or CSF biomarkers, and (3) availability of EQ-5D or VAS data. The study was approved by the Medical Ethics Review Committee of the VU University Medical Center. All patients provided written informed consent for the use of their medical data for research propose.

All participants presented as patients at the memory clinic of the Alzheimer center Amsterdam, where they received a standardized dementia diagnostic work-up, which consisted of medical history, neurological, physical and neuropsychological evaluation, MRI, laboratory tests, and lumbar puncture [11, 12]. Subsequently, clinical diagnosis (i.e., SCD, MCI or AD dementia) was made in a multi-disciplinary meeting. Patients were diagnosed with AD dementia or MCI according to the National Institute on Aging-Alzheimer’s Association (NIA-AA) criteria [13, 14]. Patients were labeled SCD when they presented with cognitive complaints, had normal clinical and cognitive test results, and did not meet the criteria for MCI, dementia or other neurologic or psychiatric conditions [15]. Annual follow-up visit included clinical assessment and neuropsychological evaluation [11, 12].

### Quality of life

During the standardized dementia diagnostic work-up and the follow-up visits between 2009 and 2018, patients were asked to complete the EQ-5D questionnaire based on the three-level version of the questionnaire (EQ-5D-3L) and/or VAS on paper [16]. In 2020, we started onlineADC, an online data collection of questionnaires related to patient-relevant outcomes (PROs), including EQ-5D five level version (EQ-5D-5L) and VAS [17]. We invited patients who had ever visited the memory clinic and their caregivers by email to complete the questionnaires in our online platform. A previous study showed that patient-reported outcome measures (PROMs) administrated on paper are comparable with questionnaires administrated on an electronic device [18].

Patients with at least one completed EQ-5D or VAS questionnaire were included in the present study. In total, we included *n* = 2170 EQ-5D questionnaires from 1140 persons (EQ-5D-3L/on paper: *n* = 1290, EQ-5D-5L/online: *n* = 880) and *n* = 2345 VAS questionnaires (*n* = 1465 on paper, *n* = 880 online). There were median (IQR) 2.0 (1.0–2.0) completed EQ-5D questionnaires per person and median (IQR) 2.0 (1.0–3.0) completed VAS questionnaires per person. The median (IQR) time between first recorded diagnosis at the memory clinic and completing the first questionnaire was 1.0 (0.0-2.0) years. The total median (IQR) follow-up time was 3.0 (2.0–3.0) years.

The EQ-5D was developed by the EuroQoL group as a standardized, non-disease-specific instrument for describing and valuing health states [19]. Patients were asked to rate their current health state in terms of five domains: mobility, self-care, usual activities, pain/discomfort and anxiety/depression. In the EQ-5D-3L version, each domain has three possible responses: no problems, some problems, or severe problems. The EQ-5D-5L has five possible responses: no problems, slight problems, moderate problems, severe problems, or unable to/extreme problems. The utility tariffs map each combination of responses on the EQ-5D to a score between 1 (perfect health) and 0 (death) and has negative values indicating a health state worse than death. The EQ-5D-5L responses were converted into an EQ-5D utilities using a Netherlands-based algorithm [20]. A “reverse crosswalk” value set was used to convert the EQ-5D-3L responses to utilities based on EQ-5D-5L values [21]. The visual analogue scale (VAS) included in the EQ-5D assesses the current health status, ranging from 0 (the worst health) to 100 (the best health).

### Amyloid status

We used amyloid-PET and CSF Aβ42 (measured at first recorded diagnosis) to determine amyloid status. Patients were categorized as amyloid-positive if they had a positive amyloid-PET scan (*n* = 164) or abnormal CSF amyloid-ß_1-42_ (Aβ42) values (*n* = 502). Patients were categorized as amyloid-negative if they had a normal amyloid-PET scan (*n* = 142) or normal CSF Aβ42 values (*n* = 332). If both amyloid-PET and CSF values were available, we used the result of the amyloid-PET scan.

CSF was obtained by lumbar puncture, collected in polypropylene tubes (Sarstedt Nurnberg, Germany) and processed according to international guidelines [22]. Before 2018, amyloid beta (Aβ42), total tau (t-tau), and phosphorylated threonine 181 (p-tau) were measured using sandwich ELISA’s (Innotest, Fujirebio, Gent, Belgium) (*n* = 633) [23]. Amyloid beta values were drift-corrected [24]. After 2018, CSF was analyzed using Elecsys (*n* = 201). CSF concentrations were considered amyloid-positive if CSF Aβ42 drift-corrected ELISA < 813 or CSF Aβ42 Elecsys < 1000 pg/ml. Amyloid-PET scans made using 3-Tesla Ingenuity TF PET/MRI, Ingenuity TF PET/CT, and Gemini TF PET/CT scanners (Philips healthcare, the Netherlands) were visually rated by an experienced nuclear medicine physician according to manufacturer guidelines. In general, images were rated as positive when unilateral binding in one or more cortical brain regions (or striatum in case of ^18^F-florbetaben or ^11^C-Pittsburgh compound B) was observed and negative when predominantly white matter uptake was seen. Amyloid-PET scans were assessed together with a T1-weighted MRI or CT-scan to assist reading in the presence of atrophy. The amyloid-PET procedure using ^18^F-florbetaben (*n* = 73), ^18^F-Florbetapir (*n* = 98), ^18^F-flutemetamol (*n* = 50), or ^11^C-Pittsburgh compound B (PiB) (*n* = 84) have been described in detail elsewhere [25, 26].

### Other variables

Follow-up time was measured in years from the first recorded diagnosis at the memory clinic to the date of EQ-5D and/or VAS was completed. The following variables were recorded during the first visit at the memory clinic: comorbidity was defined using Charlson Comorbidity Index (CCI), which was calculated based on medical history and medication use (CCI score ranges from 0 (low comorbidity) to 37 (high comorbidity)) [27]. Educational level was assessed using the Verhage scale, ranging from one (none or low educational level) to seven (high educational level: university degree) [28].Other variables we used were Mini-Mental State examination (MMSE), Rey-Auditory Verbal Learning Test (RAVLT) immediate and delayed recall, and Geriatric Depression Scale (GDS).

### Statistical analysis

Statistical analyses were performed using STATA SE version 14.0 and the figures were created in R (version 4.0.3, R Development Core Team). Normally distributed continuous variables were represented as means with standard deviations (SD), non-normally distributed continuous variables as medians with interquartile ranges (IQR), and categorical variables as the number of cases with percentages. We analyzed group differences using *t*-tests and ANOVAs for normally distributed continuous variables, Mann-Whitney and Kruskal-Wallis tests for non-normally distributed continuous variables, and chi-squared tests for categorical variables.

First, we used linear mixed-effects models (LMM) with random intercepts to compare QoL trajectories based on both EQ-5D utilities and VAS scores between amyloid-positive and amyloid-negative patients in the SCD or MCI stage. SCD and MCI patients were analyzed separately. We included terms for amyloid status and the interaction between time and amyloid status as determinants in the models. As a result, the main effect of amyloid status represents the average difference between amyloid-positive and amyloid-negative patients at baseline and the interaction effect represents the average difference in QoL over time between amyloid-positive and amyloid-negative patients. Second, we used LMM models with random intercepts to compare QoL trajectories between amyloid-positive SCD, MCI, and dementia groups using interaction terms between follow-up time and diagnosis groups. In these models, the AD dementia group was used as the reference category. In a post hoc analysis, we used LMM to compare cognitive functioning (MMSE and RAVLT) over time between amyloid-positive and amyloid-negative SCD or MCI patients.

We adjusted LMM models for EQ-5D and VAS for two confounder sets: model 1 was adjusted for age and sex, and model 2 was additionally adjusted for CCI, education, EQ-5D version (3 or 5 level; EQ-5D only). We additionally adjusted for GDS in model 3 in the models comparing QoL between amyloid-positive and amyloid-negative SCD or MCI patients.

## Results

### Patient characteristics

Patient characteristics are summarized in Table 1. Compared to amyloid-negative SCD patients, amyloid-positive SCD patients were older, more often female, had a lower MMSE score at the first visit, and had a higher comorbidity score. Amyloid-positive MCI patients were on average older, more often female, and had a lower GDS score and a lower RAVLT delayed recall score than amyloid-negative MCI patients. When we compared syndrome diagnosis groups across the AD spectrum, we observed that patients with dementia due to AD had a lower educational level, had a lower MMSE score, had a higher comorbidity score, and had a lower RAVLT immediate and delayed recall than amyloid-positive SCD and MCI patients.

**Table 1:** Patient characteristics

Baseline characteristics	SCD			MCI			Dementia	Amyloid-positive SCD, MCI, dementia^a^
	Amyloid- positive (***n*** = 105)	Amyloid-negative (***n*** = 342)	***p***-value	Amyloid- positive (***n*** = 144)	Amyloid- negative (***n*** = 132)	***p***-value	Amyloid- positive (***n*** = 417)	***p***-value
**Age, mean years (SD)**	64.6 (6.6)	60.7 (6.2)	< 0.001*	65.9 (6.6)	63.3 (7.3)	0.002*	64.4 (7.0)	0.08
**Female,** ***n*** **(%)**	50 (47)	129 (38)	0.07	65 (45)	25 (19)	< 0.001*	213 (51)	0.44
**Education Verhage, mean (SD)**	5.6 (1.2)	5.5 (1.1)	0.38	5.4 (1.3)	5.1 (1.2)	0.06	5.1 (1.2)	< 0.001*
**MMSE, median (IQR)**	28 (27–29)	29 (27–30)	0.02*	27 (25–28)	27 (26–28)	0.24	23 (19–25)	< 0.001*
**CCI, mean (SD)**	2.4 (1.1)	2.1 (1.2)	0.05	2.7 (1.2)	2.8 (1.7)	0.64	3.4 (1.2)	< 0.001*
**GDS, median (IQR)**	2 (1–4)	2 (1–4)	0.59	2 (1–4)	3 (2–6)	0.001*	2 (1–4)	0.82
**RAVLT** **Immediate recall, mean (SD)**	41.9 (8.3)	42.3 (9.3)	0.67	31.0 (7.2)	31.5 (7.8)	0.60	23.3 (8.8)	< 0.001*
**RAVLT** **Delayed recall, mean (SD)**	8.1 (3.0)	8.6 (3.0)	0.16	3.4 (2.5)	4.4 (2.7)	0.001*	2.1 (2.6)	< 0.001*

*Statistically significant based on *p* < 0.05

^a^See Additional file 1 for post hoc analysis

*SCD* subjective cognitive decline, *MCI* mild cognitive impairment, *MMSE* mini-mental state examination, *CCI* Charlson Comorbidity Index, *GDS* Geriatric Depression Scale, *RAVLT* Rey-Auditory Verbal Learning

*P*-values were obtained using independent samples *t*-tests or ANOVA for normally distributed continuous variables (mean (SD)), Mann-Whitney two samples tests for non-normally distributed continuous variables (median (IQR)), and chi-square tests for categorical variables (*n*(%))

### Quality of life trajectories in amyloid-positive and amyloid-negative patients

**Fig. 1:**
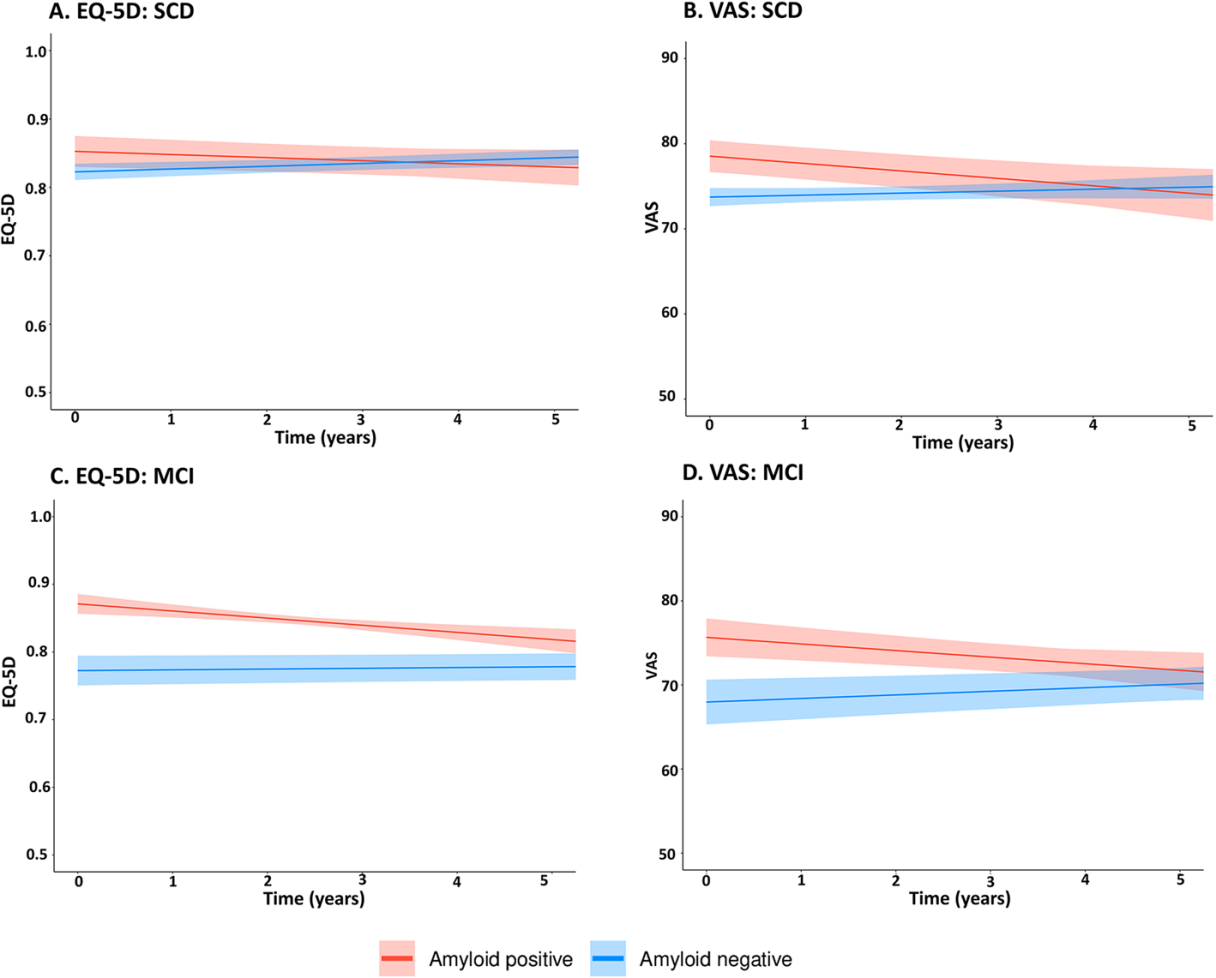
EQ-5D and VAS trajectories in amyloid positive and amyloid negative SCD and MCI patients. The lines represent estimated group trajectories of unadjusted QoL scores over time in years with 95% confidence intervals. EQ-5D, the European Quality of Life-5 Dimensions; VAS, the visual analogue scale; SCD, subjective cognitive decline; MCI, mild cognitive impairment

Table 2 shows the differences in the QoL between amyloid-positive and amyloid-negative SCD and MCI patients. For SCD, LMM revealed no baseline differences in EQ-5D utility between amyloid-positive and amyloid-negative patients, but there were differences in EQ-5D over time between amyloid-positive and amyloid-negative patients (*p* for interaction < 0.05). EQ-5D of amyloid-positive patients with SCD decreased over time, while EQ-5D of amyloid-negative SCD patients remained stable (Table 2 and Fig. 1A). When we evaluated VAS, we found that amyloid-positive patients had a higher VAS at baseline but showed a steeper decline over time than amyloid-negative patients. The VAS score of amyloid-positive SCD patients decreased over time, while by contrast, the VAS score of amyloid-negative patients increased over time (Table 2 and Fig. 1B). For example, VAS at baseline of amyloid-positive SCD patients was 4.03 lower compared to amyloid-negative SCD patients and VAS decreased with 1.08 per year compared to the VAS score of amyloid-negative patients.

**Table 2:** Differences in quality of life trajectories between amyloid-positive and amyloid-negative SCD and MCI patients

		EQ-5D utilities				VAS			
		Unadjusted	Model 1	Model 2	Model 3	Unadjusted	Model 1	Model 2	Model 3
		*Β* (SE)	*Β* (SE)	*Β* (SE)	*Β* (SE)	*Β* (SE)	*Β* (SE)	*Β* (SE)	*Β* (SE)
**SCD**	*Aβ* positive	0.03 (0.02)	0.03 (0.02)	0.03 (0.02)	0.03 (0.02)	4.81 (2.02)*	4.57 (2.08)*	4.03 (2.05)*	4.06 (1.96)*
	Time * *Aβ* positive	− 0.01 (0.004)*	− 0.01 (0.004)*	− 0.01 (0.004)*	− 0.01 (0.004)*	− 1.10 (0.45)*	− 1.08 (0.45)*	− 1.08 (0.45)*	− 0.96 (0.45)*
**MCI**	*Aβ* positive	0.10 (0.02)*	0.10 (0.02)*	0.09 (0.02)*	0.07 (0.21)*	7.68 (2.25)*	7.65 (2.32)*	6.01 (2.30)*	4.34 (2.32)
	Time * *Aβ* positive	− 0.01 (0.004)*	− 0.01 (0.004)*	− 0.01 (0.004)*	− 0.01 (0.004)*	− 1.21 (0.44)*	− 1.19 (0.44)*	− 1.12 (0.44)*	− 1.07 (0.44)*

Model 1: adjusted for age and sex

Model 2: additionally adjusted for comorbidity score, education, squared education (only for EQ-5D models in MCI patients) and EQ-5D scale (EQ-5D only)

Model 3: additionally adjusted for GDS

*p < 0.05

Of note: main effect of amyloid status represents the average difference between amyloid-positive and amyloid-negative patients at baseline; interaction effect represents the difference in QoL over time between amyloid-positive and amyloid-negative patients

*SCD* subjective cognitive decline, *MCI* mild cognitive impairment, *Aβ positive* amyloid positive, *SE* standard error, *GDS* Geriatric Depression Scale

For MCI, we found differences in EQ-5D and VAS between amyloid-positive and amyloid-negative patients (Table 2). Amyloid-positive MCI patients had a higher QoL at baseline compared to amyloid-negative patients. Whereas the EQ-5D of amyloid-positive MCI patients decreased over time, the EQ-5D of amyloid-negative patients remained stable over time (Table 2 and Fig. 1C). The VAS score of amyloid-positive MCI patients decreased over time, while the VAS score of amyloid-negative patients increased over time (Table 2 and Fig. 1D). After additionally adjusted model 2 for GDS at baseline, the baseline difference between amyloid-negative an amyloid-positive MCI patients in VAS disappeared (Table 2).

Compared to model 1, the observed effects for EQ-5D did not change in both SCD and MCI patients. The observed baseline differences in VAS were somewhat attenuated after additional adjustment in model 2 for both SCD and MCI patients but remained significant.

### Quality of life trajectories in diagnosis groups

**Fig. 2:**
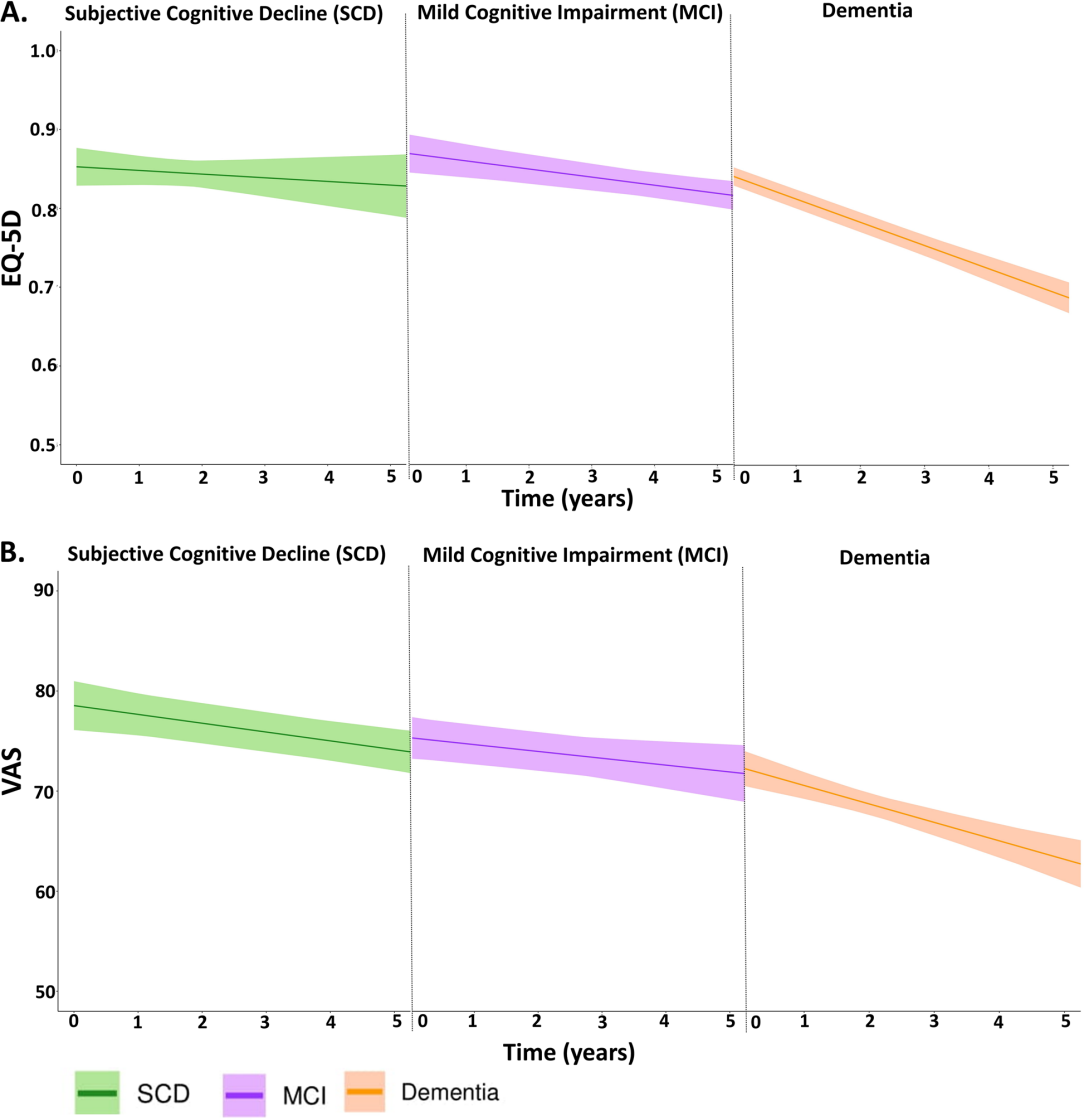
EQ-5D and VAS trajectories over time in amyloid-positive SCD, MCI and dementia patients. The lines represent estimated group trajectories of unadjusted QoL scores over time in years with 95% confidence intervals. EQ-5D, the European Quality of Life-5 Dimensions; VAS, the visual analogue scale

Table 3 shows the differences in the QoL trajectories along the Alzheimer continuum, of amyloid-positive patients with dementia (reference group) and amyloid-positive patients with MCI or SCD. LMM revealed no baseline differences in EQ-5D or VAS between syndrome diagnosis groups. However, there were interaction effects of syndrome diagnosis groups by time, as patients with dementia showed a steeper decline than patients with SCD or MCI on both measures of QoL (Table 3 and Fig. 2). Compared to model 1, the observed differences at baseline decreased, but the differences in the trajectories of QoL between the groups did not change in model 2 compared to model 1.

**Table 3:** Differences in quality of life trajectories between amyloid-positive patients with SCD, MCI, and dementia

	EQ-5D utilities			VAS		
	Unadjusted	Model 1	Model 2	Unadjusted	Model 1	Model 2
	*Β* (SE)	*Β* (SE)	*Β* (SE)	*Β* (SE)	*Β* (SE)	*Β* (SE)
SCD	0.02 (0.02)	0.02 (0.02)	0.001 (0.02)	6.52 (2.15)*	6.45 (2.15)*	4.20 (2.31)
MCI	0.04 (0.02)	0.03 (0.02)	0.02 (0.02)	3.38 (1.93)	3.21 (1.94)	1.51 (2.05)
Dementia	Ref	Ref	Ref	Ref	Ref	Ref
Time * SCD	0.02 (0.005)*	0.02 (0.005)*	0.03 (0.005)*	0.94 (0.54)**	0.95 (0.54)**	0.97 (0.54)**
Time * MCI	0.02 (0.005)*	0.02 (0.005)*	0.02 (0.005)*	1.13 (0.51)*	1.14 (0.51)*	1.14 (0.51)*
Time * dementia	Ref	Ref	Ref	Ref	Ref	Ref

Model 1: adjusted for age, squared age (EQ-5D only), and sex

Model 2: additionally adjusted for comorbidity score, education, and EQ-5D scale (EQ-5D only)

*p < 0.05

**p < 0.10 for interaction term

Of note: main effect of syndrome diagnosis groups represents the average difference between SCD vs. dementia and MCI vs. dementia at baseline; interaction effect represents the difference in QoL over time between SCD vs. dementia and MCI vs. dementia

*SCD* subjective cognitive decline, *MCI* mild cognitive impairment, *Aβ positive* amyloid positive, *SE* standard error

## Discussion

In this longitudinal study, we compared trajectories of EQ-5D and VAS between amyloid-positive and amyloid-negative patients along the Alzheimer’s disease (AD) continuum of cognitively normal to dementia. Although initially reporting higher QoL, amyloid-positive SCD and MCI patients showed a steeper decline over time in EQ-5D and VAS than amyloid-negative patients. In addition, when we evaluated the full continuum of AD, QoL decreased at a faster rate in patients with dementia compared to amyloid-positive patients with SCD or MCI.

A recent cross-sectional study among biomarker-confirmed AD patients in the SCD and MCI stages showed no significant difference in EQ-5D score between amyloid-positive and amyloid-negative SCD patients and a somewhat counter-intuitively higher EQ-5D score in amyloid-positive MCI patients compared to amyloid-negative MCI [10]. We confirmed these results and we also observed higher GDS in amyloid-negative MCI patients compared to amyloid-positive patients (Table 1). The more depressive symptoms at baseline may also explain the lower QoL at baseline in amyloid-negative MCI patients. After we additionally adjusted for GDS at baseline in model 3, the baseline difference in VAS disappeared (Table 2). We additionally showed that longitudinal data are essential to understand the impact of amyloid on QoL. Despite a similar baseline QoL, the EQ-5D of amyloid-positive SCD and MCI patients decreased at a faster rate over time than the EQ-5D of amyloid-negative SCD or MCI patients. The observed decrease in QoL in amyloid-positive individuals could be attributable to continuing disease progression, with (subtly) increasing cognitive and functional decline, or the observed decrease could be due to uncertainty of an amyloid-positive result. Additional file 2 contains results that confirm increased cognitive decline in amyloid-positive patients, but more research is needed to gain a detailed understanding of the underlying factors that explain the decline in QoL in amyloid-positive patients. By contrast, we found that QoL improves (VAS) or remained stable (EQ-5D) over time in amyloid-negative individuals with SCD or MCI, which could be due to relief or reassurance that AD is not the underlying cause of their complaints and/or improvement of the condition that initially caused their signs and symptoms (e.g., sleep problems, depressive symptoms).

To date, most studies on QoL in AD were based on cross-sectional data mostly in the dementia stage and lacking biomarker support of diagnosis [6]. Our paper adds to the existing literature by providing insight into the trajectories for biomarker confirmed AD over a mean follow-up time of 3 years. In addition, there is a lack of studies on QoL in the pre-dementia SCD and MCI stages. We included a large sample of patients with diagnoses ranging from SCD, MCI to AD dementia and showed a steeper decline in QoL in dementia than patients with SCD or MCI.

Knowledge about the natural QoL trajectories along the complete AD continuum can be used to evaluate the potential impact of future disease-modifying treatments on QoL. However, there are a number of challenges to measure QoL in AD patients [29]. Especially in a later stage, it is difficult for AD patients to indicate their QoL due to cognitive decline. In addition, it is questionable whether the available QoL scales accurately reflect QoL in AD. Nevertheless, governments and health insurance companies base the decision to reimburse treatments on the costs per quality-adjusted life year gained from treating AD patients with the new treatments [30, 31]. Therefore, QoL is an important outcome measure when evaluating the effectiveness of treatment for AD. In addition, disease-modifying treatments increasingly focus on the pre-dementia stages in AD to delay dementia onset and its associated decrease in QoL. Therefore, it is important to have insight into QoL across the entire trajectory of the disease. The results from this study can be used to inform future studies that aim to demonstrate an effect of a treatment on QoL.

A limitation of this study is the potential selective drop-out of patients in a more advanced disease stage. Therefore, the results presented in this paper may underestimate the true decline in QoL in the course of AD. Another potential limitation is that we used two different EQ-5D versions (EQ-5D-3L and EQ-5D-5L). However, we converted the EQ-5D-3L responses to EQ-5D utilities based on EQ-5D-5L values. In addition, we adjusted the models for EQ-5D version. Finally, EQ5D included domains (i.e., mobility, self-care, pain) may not be affected by AD in early stages AD, as patients with SCD and MCI mainly have cognitive complaints and not yet any physical or functional consequences. We measured QoL in two different ways (EQ5D and VAS), and we found no difference in EQ5D at baseline between amyloid-positive and amyloid-negative SCD. However, we did find a difference in VAS at baseline between these groups. Therefore, VAS may be more sensitive to detect differences in QoL in early AD, as it assesses overall health status.

In conclusion, the trajectories EQ-5D and VAS two measures of QoL showed steeper decline over time in amyloid-positive SCD and MCI patients compared to amyloid-negative patients. Moreover, QoL decreased at a faster rate in patients with dementia compared to amyloid-positive SCD or MCI patients. Knowledge of QoL trajectories along the full trajectory of AD is essential for the evaluation of the effect on QoL of (future) treatments for AD.

## Supplementary Information

**Additional file 1.** Post hoc analysis of patient characteristics in amyloid-positive patients.

**Additional file 2.** Post hoc analysis of differences in MMSE and RAVLT between amyloid-positive and amyloid-negative SCD and MCI patients.

## Data Availability

The datasets used and/or analyzed during the present study are available from the corresponding author on reasonable request.

## Abbreviations

AD: Alzheimer’s disease
SCD: Subjective cognitive decline
MCI: Mild cognitive impairment
VAS: Visual analog scale
EQ-5D: European Quality of Life-5 Dimensions
QoL: Quality of life
ADC: Amsterdam Dementia Cohort
PROs: Patient-relevant outcomes
MMSE: Mini-mental state examination
CCI: Charlson Comorbidity Index
