# Supplementary material for: A longitudinal study on quality of life along the spectrum of Alzheimer’s disease
Source: Alzheimers Res Ther. 2022 Sep 15;14:132. doi: 10.1186/s13195-022-01075-8 (PMC9476356; doi:10.1186/s13195-022-01075-8)
Supplement: Supplementary file 1 — Additional file 1. Post hoc analysis of patient characteristics in amyloid-positive patients. [file 13195_2022_1075_MOESM1_ESM.docx]

**Additional file 1.** Post hoc analysis of patient characteristics in amyloid-positive patients

| **Baseline characteristics** | **SCD vs. MCI†** | **SCD vs. dementia†** | **MCI vs. dementia†** |
| --- | --- | --- | --- |
|  | ***p*-value** | ***p*-value** | ***p*-value** |
| **Age, mean years (SD)** | 0.13 | 0.82 | 0.03 |
| **Female, n (%)** | 0.70 | 0.53 | 0.22 |
| **Education Verhage, mean (SD)** | 0.09 | <0.001* | 0.01* |
| **MMSE, median (IQR)** | <0.001* | <0.001* | <0.001* |
| **CCI, mean(SD)** | 0.07 | <0.001* | <0.001* |
| **GDS, median (IQR)** | 0.73 | 0.61 | 0.30 |
| **RAVLT**  **Immediate recall, mean (SD)** | <0.001* | <0.001* | <0.001* |
| **RAVLT**  **Delayed recall, mean (SD)** | <0.001* | <0.001* | <0.001* |
| *Statistically significant based on Bonferroni-corrected p-value of <0.017  †Amyloid-positive patients  SCD= subjective cognitive decline, MCI=mild cognitive impairment, AD=Alzheimer’s disease, MMSE=mini-mental state examination, CCI=charlson comorbidity utilities, GDS=Geriatric Depression Scale, RAVLT=Rey-Auditory Verbal Learning | | | |
