# Supplementary material for: A longitudinal study on quality of life along the spectrum of Alzheimer’s disease
Source: Alzheimers Res Ther. 2022 Sep 15;14:132. doi: 10.1186/s13195-022-01075-8 (PMC9476356; doi:10.1186/s13195-022-01075-8)
Supplement: Supplementary file 2 — Additional file 2. Post hoc analysis of differences in MMSE and RAVLT between amyloid-positive and amyloid-negative SCD and MCI patients. [file 13195_2022_1075_MOESM2_ESM.docx]

**Additional file 2.** Post hoc analysis of differences in MMSE and RAVLT between amyloid-positive and amyloid-negative SCD and MCI patients

|  |  |  | **MMSE** | |  | **RAVLT**  **Immediate recall** | |  | **RAVLT**  **Delayed recall** | |
| --- | --- | --- | --- | --- | --- | --- | --- | --- | --- | --- |
|  |  | **Unadjusted** | **Model 1** | **Model 2** | **Unadjusted** | **Model 1** | **Model 2** | **Unadjusted** | **Model 1** | **Model 2** |
|  |  | *Β* (SE) | *Β* (SE) | *Β* (SE) | *Β* (SE) | *Β* (SE) | *Β* (SE) | *Β* (SE) | *Β* (SE) | *Β* (SE) |
| **SCD** | *Aβ* positive | -0.19 (0.17) | -0.16 (0.18) | -0.22 (0.16) | -0.49 (1.05) | -0.26 (1.06) | -0.58 (0.96) | -0.49 (0.33) | -0.30 (0.34) | -0.51 (0.32) |
|  | Time * *Aβ* positive | -0.25 (0.04)* | -0.25 (0.04)* | -0.25 (0.04)* | -1.84 (0.20)* | -1.84 (0.20)* | -1.83 (0.20)* | -0.64 (0.06)* | -0.64 (0.06)* | -0.64 (0.06)* |
| **MCI** | *Aβ* positive | -0.35 (0.31) | -0.37 (0.32) | -0.51 (0.32) | -1.58 (0.94) | -1.73 (0.96) | -2.11 (0.98)* | -1.33 (0.31)* | -1.16 (0.32)* | -1.15 (0.33)* |
|  | Time * *Aβ* positive | -0.68 (0.08)* | -0.68 (0.08)* | -0.68 (0.08)* | -1.45 (0.22)* | -1.45 (0.22)* | -1.45 (0.22)* | -0.38 (0.06)* | -0.38 (0.07)* | -0.38 (0.07)* |
| Model 1: adjusted for age and sex.  Model 2: additionally adjusted for comorbidity score, education  *p<0.05  Of note: main effect of amyloid status represents the average difference between amyloid-positive and amyloid-negative patients at baseline; interaction effect represents the difference in MMSE or RAVLT over time between amyloid-positive and amyloid-negative patients.  SCD= subjective cognitive decline, MCI=mild cognitive impairment; MMSE= Mini-mental state examination; RAVLT=Rey-Auditory Verbal Learning Test; *Aβ* positive=amyloid positive, SE=standard error | | | | | | | | | | |
